# Dosimetric investigation of couch rotation angles in non-coplanar VMAT plans for lung cancer SBRT

**DOI:** 10.3389/fonc.2024.1454676

**Published:** 2024-12-24

**Authors:** Weiqiang Ye, Housheng Wang, Zhenzhen Wei, Wei Zhang, Chaojun Yu, Dawei Zhang, Shida Su, Wen Qin, Kai Hu, Bo Li

**Affiliations:** ^1^ Department of Radiation Oncology, The First Affiliated Hospital of Guangxi Medical University, Nanning, Guangxi, China; ^2^ Key Laboratory of Early Prevention and Treatment for Regional High Frequency Tumor (Guangxi Medical University), Ministry of Education, Nanning, Guangxi, China; ^3^ Guangxi Key Laboratory of Immunology and Metabolism for Liver Diseases, Nanning, Guangxi, China; ^4^ State Key Laboratory of Targeting Oncology, Guangxi Medical University, Nanning, Guangxi, China

**Keywords:** couch rotation angles, non-coplanar VMAT, SBRT, lung cancer, dosimetry

## Abstract

**Background:**

This study aimed to investigate the effect of couch rotation angles on non-coplanar volumetric modulated arc therapy (ncVMAT) plan for stereotactic body radiotherapy (SBRT) in lung cancer patients and to evaluate the feasibility of clinically applying ncVMAT for SBRT.

**Methods:**

Twenty-four lung cancer patients with a single lesion eligible for SBRT were enrolled in the study. Seven dual partial-arc VMAT plans with varying couch angles were designed for every patient. These plans utilized two partial arcs, with the same first arc set at a fixed 0° couch angle in all plans. The second arc’s couch angle varies at 15° intervals, ranging from 0° to 90°. The plans are designated as C_0_, NC_15_, NC_30_, NC_45_, NC_60_, NC_75_, and NC_90_, respectively. Plan evaluation included assessment of the maximum dose (D_max_), the mean dose (D_mean_), homogeneity index (HI), conformity index (CI), and the ratio of the 50% isodose volume to the planning target volume (R50%). Dosimetric parameters for organs at risk such as the ipsilateral lung, contralateral lung, bilateral lungs, esophagus, trachea, chest wall, heart, and spinal cord were analyzed. Additionally, plan complexity-related metrics included modulation degree (MD), delivery time (DT), and monitor unit (MU) were assessed.

**Results:**

As the couch rotation angle increased, parameters such as D_max_, D_mean_, HI, CI, R50%, V_20Gy_, V_25.75Gy_ and V_30Gy_ of the ipsilateral lung and bilateral lungs, V_10Gy_ of the contralateral lung and D_mean_ of the chest wall varied, while MD, MU, and DT increased. Compared to C_0_, the D_max_, D_mean_, and HI of the planning target volume (PTV) decreased from 6728.35 ± 209.56cGy, 5743.04 ± 93.45cGy, and 0.281 ± 0.032 to 6500.48 ± 225.26cGy, 5654.81 ± 109.23cGy, and 0.245 ± 0.031, respectively, when the couch was rotated to 90°. The CI increased from 0.859 ± 0.031 to 0.876 ± 0.024. Decreases in R50% were 1.4%, 4.9%, 9%, 13.5%, 16.8%, and 18.4% for NC_15_, NC_30_, NC_45_, NC_60_, NC_75_, and NC_90_, respectively.

**Conclusions:**

In the treatment of lung cancer using SBRT, ncVMAT plans demonstrate superior dose distribution and deliver lower doses to certain OARs compared to cVMAT plans. This advantage becomes more pronounced with increasing couch rotation angles. Our study offers theoretical support for the preferential use of ncVMAT plans in lung cancer SBRT and provides empirical evidence to guide the selection of optimal couch rotation angles.

## Introduction

Lung cancer is the most commonly diagnosed malignancy and the leading cause of cancer-related mortality worldwide, accounting for approximately 12.4% of all cancer cases and contributing to a mortality rate of 18.7% (1, 2). Radiotherapy has long been a cornerstone in the treatment of lung cancer. With advancements in medical physics and radiation technology, stereotactic body radiotherapy (SBRT) has emerged as a promising treatment option for patients with inoperable early-stage non-small cell lung cancer (NSCLC) and lung metastases (3). SBRT delivers high doses of radiation with precision in a limited number of fractions, significantly enhancing local tumor control and survival compared to conventional radiotherapy. Due to its non-invasive nature and efficacy, some studies suggest that SBRT may even surpass surgery in effectiveness, though further validation is required (4–8).

Linear accelerator-based SBRT techniques include three-dimensional conformal radiotherapy, intensity-modulated radiotherapy, and volumetric-modulated arc therapy (VMAT). VMAT delivers radiation while the gantry rotates continuously, enabling simultaneous gantry rotation, multileaf collimator (MLC) movement, and dose rate modulation. This approach provides superior dose conformality, improved homogeneity, and shorter treatment times for patients (9, 10). Currently, coplanar VMAT (cVMAT) is the most widely used technique for SBRT due to its simplicity. However, non-coplanar techniques are less frequently employed due to the complexity and uncertainty introduced by couch rotation (11). Non-coplanar VMAT (ncVMAT), which utilizes multiple arcs from different planes relative to the patient, offers distinct advantages in SBRT by reducing beam overlap, increasing the number of beam incidence directions, and achieving steeper dose fall-off outside the target volume (12). A previous study (13) demonstrated that ncVMAT with 15° opposite couch kicks significantly reduced V30 and V40 doses to the chest wall without increasing lung doses, compared to cVMAT. Additionally, Ishii et al. (14) compared three beam arrangement strategies for SBRT of centrally located lung tumors: two non-coplanar partial arcs, two coplanar partial arcs, and one coplanar full arc. Their findings indicated that ncVMAT plans resulted in reduced irradiation to mediastinal organs at risk.

Non-coplanar VMAT may represent a more effective approach for SBRT; however, the relationship between couch rotation angles and dosimetric distribution remains unclear. This study aims to investigate the impact of couch rotation angles on ncVMAT plans for SBRT in lung cancer, analyze the dosimetric benefits of varying couch rotation angles, and identify the optimal couch rotation angle for SBRT.

## Methods

### Patient selection and simulation

Twenty-four patients (eighteen men and six women) treated with SBRT lung cancer at the First Affiliated Hospital of Guangxi Medical University were retrospectively included in this study. The patients had an average age of 58.5 ± 12.2 years, ranging from 33 to 88 years. A detailed summary of patient characteristics is shown in **Table 1**. This study was approved by the Institutional Review Board of the First Affiliated Hospital of Guangxi Medical University.

**Table 1:** The characteristics of 24 patients.

Patient^#^	Sex	Age	Location	Tumor size (cm)	PTV volume (cc)
1	Female	56	Right Upper	2.86	18.92
2	Male	74	Left Upper	2.17	11.01
3	Male	49	Left Upper	2.01	11.9
4	Male	88	Right Upper	3.02	24.08
5	Male	33	Right Upper	2.29	9.43
6	Male	51	Right Mid	4.94	82.24
7	Female	49	Left Lower	2.67	16.68
8	Male	57	Left Upper	4.29	59.03
9	Male	61	Right Upper	1.65	12.01
10	Male	57	Left Lower	3.93	34.32
11	Male	70	Left Upper	5.18	56.33
12	Male	64	Left Lower	4.14	52.47
13	Male	51	Left Lower	4.56	25.65
14	Male	52	Right Upper	1.74	6.95
15	Male	42	Right Upper	1.69	7.69
16	Female	60	Left Upper	2.56	16.31
17	Male	71	Left Upper	2.85	20.05
18	Male	37	Left Lower	2.42	12.89
19	Female	69	Left Lower	2.47	14.32
20	Female	57	Left Upper	1.97	8.11
21	Female	62	Left Upper	3.58	38.66
22	Male	66	Right Upper	4.67	65.95
23	Male	67	Left Upper	1.98	7.41
24	Male	61	Left Upper	4.18	38.21

"#" means number (No.).

Each patient underwent a four-dimensional CT simulation on a Philips Big Bore Brilliance CT Simulator (Philips Healthcare, USA) equipped with a Philips Bellows Belt to monitor the breathing trace. Immobilization was achieved using a CIVCO SBRT device (CIVCO, USA) along with a customized vacuum cushion. To keep the arms out of the treatment fields, an arm shuttle was positioned above the trunk, with the arms raised overhead. A compression belt was also applied to the abdominal region to minimize internal organ motion during breathing cycles. Computed tomography (CT) images were acquired across 10 breathing phases.

### Contouring

Following CT simulation, images were imported into the Elekta Monaco 5.11 Treatment Planning System (Elekta, UK) for contour delineation. Maximum and average intensity projection datasets were generated across all 10 respiratory phases. Planning objectives, target volumes, and critical structures were defined based on the ICRU 83 guidelines and RTOG 0813 protocol (14, 15). The internal target volume (ITV) was contoured on the maximum intensity projection by a radiation oncologist. A uniform 5-mm isotropic margin was applied to delineate the planning target volume (PTV). Contoured organs at risk (OARs) included the ipsilateral lung, contralateral lung, bilateral lungs, heart, esophagus, trachea, chest wall, and spinal cord.

### Treatment planning

The Monaco 5.11 Treatment Planning System, which underwent clinical acceptance testing and periodic quality assurance, was employed for VMAT planning. Dose calculations were performed using the X-ray Voxel Monte Carlo algorithm with a 2-mm calculation grid, ensuring high computational accuracy. The linear accelerator (Versa HD, Elekta) was equipped with 80 pairs of 5-mm Agility MLCs, providing a maximum irradiation field of 40 × 40 cm². A 6-MV flattening filter-free photon beam was utilized, delivering a maximum dose rate of 1400 monitor units (MU) per minute, effectively reducing treatment time.

**Figure 1:**
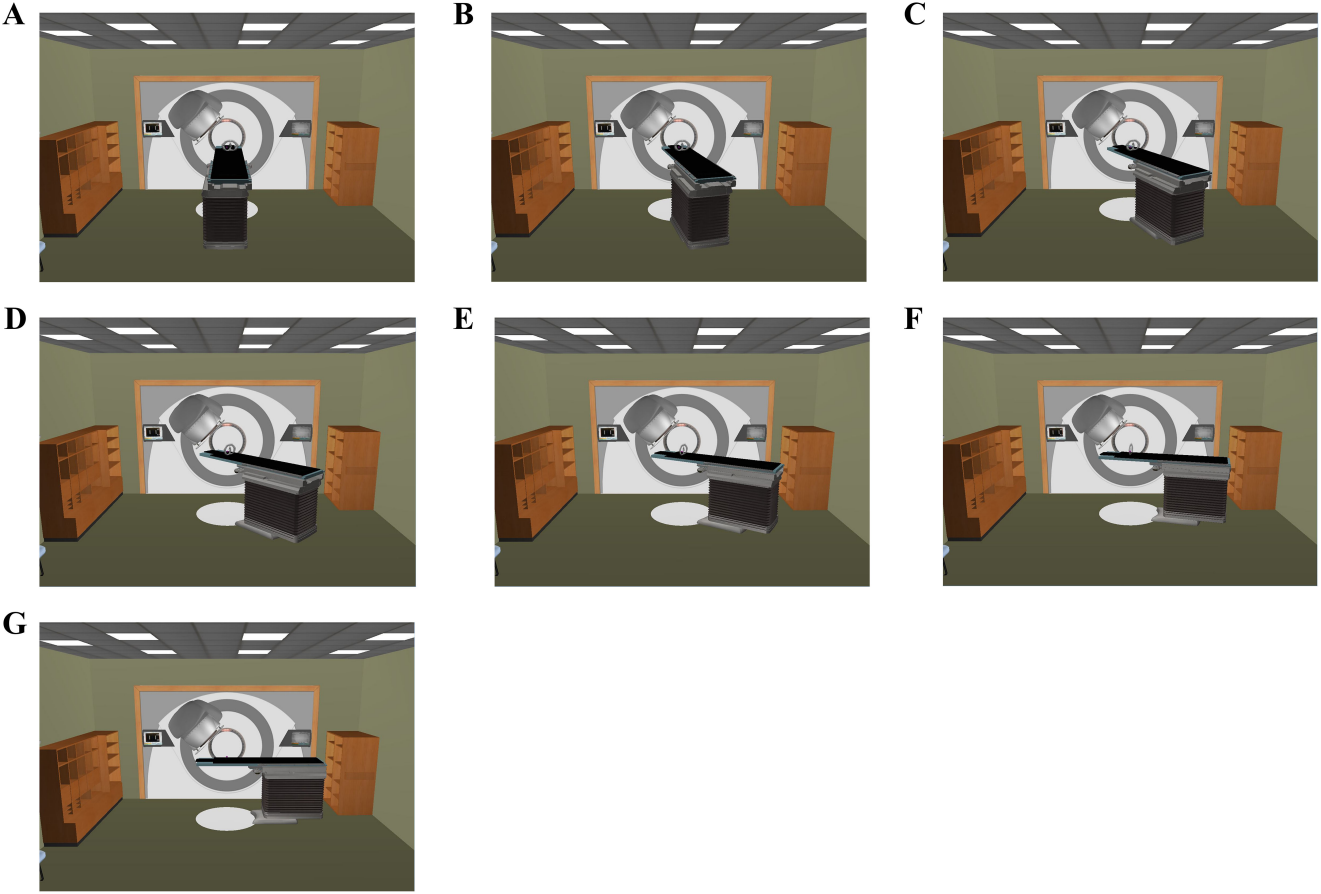
Room’s eye view of the couch rotation angle of the 2^nd^ partial arc [**(A)** 0°; **(B)** 15°; **(C)** 30°; **(D)** 45°; **(E)** 60°; **(F)** 75°; **(G)** 90°].

For each patient, an initial cVMAT treatment plan (designated as C0) was developed to meet clinical requirements using two partial coplanar arcs with the couch fixed at 0°. The first arc spanned 240°, rotating clockwise from 180° to 60°for right-sided lesions and from 300° to 180°for left-sided lesions, thereby minimizing dose exposure to the contralateral lung. The second arc spanned 90°, rotating clockwise from 315° to 45°, ensuring consistency with noncoplanar plans and reducing the risk of gantry collisions during noncoplanar treatment scenarios (**Figure 1A**). Subsequently, six ncVMAT treatment plans with varying couch rotation angles for the second arc were created by a single medical physicist, resulting in a total of seven treatment plans per patient and 168 plans overall. The first arc remained unchanged, while the couch rotation angle for the second arc was adjusted to 15°, 30°, 45°, 60°, 75°, and 90°, designated as NC15, NC30, NC45, NC60, NC75, and NC90, respectively (**Figures 1B–G**). The specific couch rotation angles for the VMAT plans’ partial arcs are detailed in **Table 2**.

**Table 2:** Couch rotation angles of two partial arcs used in the VMAT.

	C_0_	NC_15_	NC_30_	NC_45_	NC_60_	NC_75_	NC_90_
1^st^ partial Arc	0°	0°	0°	0°	0°	0°	0°
2^nd^ partial Arc	0°	15°	30°	45°	60°	75°	90°

All treatment plans prescribed a total dose of 50 Gy delivered in five fractions (10 Gy per fraction). A maximum dose exceeding 120% of the prescription dose to the PTV was deemed acceptable. To minimize planning bias, a consistent and standardized template of PTV and OAR objectives was applied uniformly across all plans without additional manual optimization. Dose constraints for OARs are summarized in **Table 3** (16–19), and the objective functions used during plan optimization are detailed in **Table 4**. Plans were considered acceptable if they adhered to the RTOG 0813 protocol, with no major deviations. Optimization was performed iteratively by adjusting objective parameters to ensure nearly 95% PTV coverage while meeting OAR constraints. Final plans were normalized to ensure that 95% of the PTV received 100% of the prescribed dose, with normalization adjustments limited to less than 1%.

**Table 3:** Dose constraints for organs at risk.

OAR	Constraints		OAR	Constraints	
bilateral Lungs	V_20_<10%	D_mean_<8 Gy	Trachea	D_max_<50 Gy	V_45_<5 cc
Heart	D_max_<52.5 Gy	V_32_<15 cc	Spinal cord	D_max_<28 Gy	V_22_<0.35 cc
Esophagus	D_max_<38 Gy	V_32.5_<5 cc	Chest wall	V_50_<2 cc	

OAR, organs at risk; D_max_, maximum dose to the planned target volume

**Table 4:** Objective functions used in treatment plan optimization.

Objective name	Objective function	Objective parameters
PTV	Target EUD	Prescription=5000cGy with 0.5 Cell Sensitivity
	Underdose DVH	90% of 5000cGy
Body	Maximum Dose	6000cGy over all voxels in volume
	Maximum Dose	5000cGy at Shrink Margin=0
	Maximum Dose	2500cGy at Shrink Margin=1~1.6cm

### Plan evaluation

Following retrospective treatment planning for each patient, PTV coverage was assessed using isodose line analysis and dose-volume histograms (DVH). Key evaluation parameters included maximum dose (Dmax), mean dose (Dmean), conformity index (CI), R50% (gradient index, GI), and homogeneity index (HI). Dmax and Dmean represented the highest and average doses delivered to the PTV, respectively. The HI and CI served as dosimetric indicators to evaluate dose distribution homogeneity and conformity of dose coverage to the target volume in each planto 1 indicated higher conformity of dose to the target, while a lower HI value signified greater dose homogeneity. Intermediate-dose conformity was assessed by R50% (GI), which quantified dose fall-off outside the target volume and was defined as the ratio of the 50% isodose volume to the PTV. A lower R50% indediate-dose conformity and a steeper dose fall-off outside the target region.

Dose metrics across plans were compared, including the mean dose, V5Gy, V10Gy, V20Gy, V25.75Gy, and V30Gy for the ipsilateral lung and both lungs combined; mean dose, V5Gy, and V10Gy for the contralateral lung; and maximum and mean doses for the esophagus, trachea, chest wall, heart, and spinal cord. Plan complexity-related metrics included evaluated included modulation degree (MD), monitor units (MU), and delivery time (DT). DT represented the estimated beam-on time calculated by the treatment planning system (TPS), excluding setup and couch movement time. MD was used to assess plan complexity in achieving the desired dose distribution. MD is an indication of complexity of the fluence maps used in the plan. The formula for MD is computed for all beams or sequences. It is defined as follows: MD = Total MU/[Sum of (Segment Area × Segment Mu/Total Beam Area)].

### Patient-specific quality assurance

Patient-specific quality assurance (PSQA) was conducted to assess the accuracy of dose delivery for these VMAT treatments. Dose distribution measurements were performed using the OCTAVIUS 4D Modular Phantom combined with the 2D liquid-filled ion chamber array, OCTAVIUS 1000 SRS (PTW, Freiburg, Germany). The 1000 SRS array comprises 977 ion chambers, each measuring 2.3 mm × 2.3 mm × 0.5 mm, within an 11 cm × 11 cm field, providing high resolution at 2.5 mm in the central area and 5 mm in the peripheral area.

The OCTAVIUS 4D Modular Phantom, fitted with the 1000 SRS array, rotates synchronously with the LINAC gantry via an inclinometer. Pre-treatment verification was completed for all treatment plans. Planar dose measurements were taken, and the three-dimensional dose distribution was reconstructed using VeriSoft 7.1 software (PTW, Freiburg, Germany). The measured dose was then compared to the planned dose, as calculated in the TPS, using the gamma index (20). A 3D gamma analysis with 2%/2mm criteria was performed in VeriSoft 7.1, with a passing rate of over 90% considered acceptable.

### Statistical analysis

All data analyses were conducted using SPSS software (IBM, NY, USA), with results presented as mean ± standard deviation (SD). Statistical analysis was performed using the nonparametric Jonckheere-Terpstra test, and a p-value of less than 0.05 was considered statistically significant.

## Results

**Figure 2:**
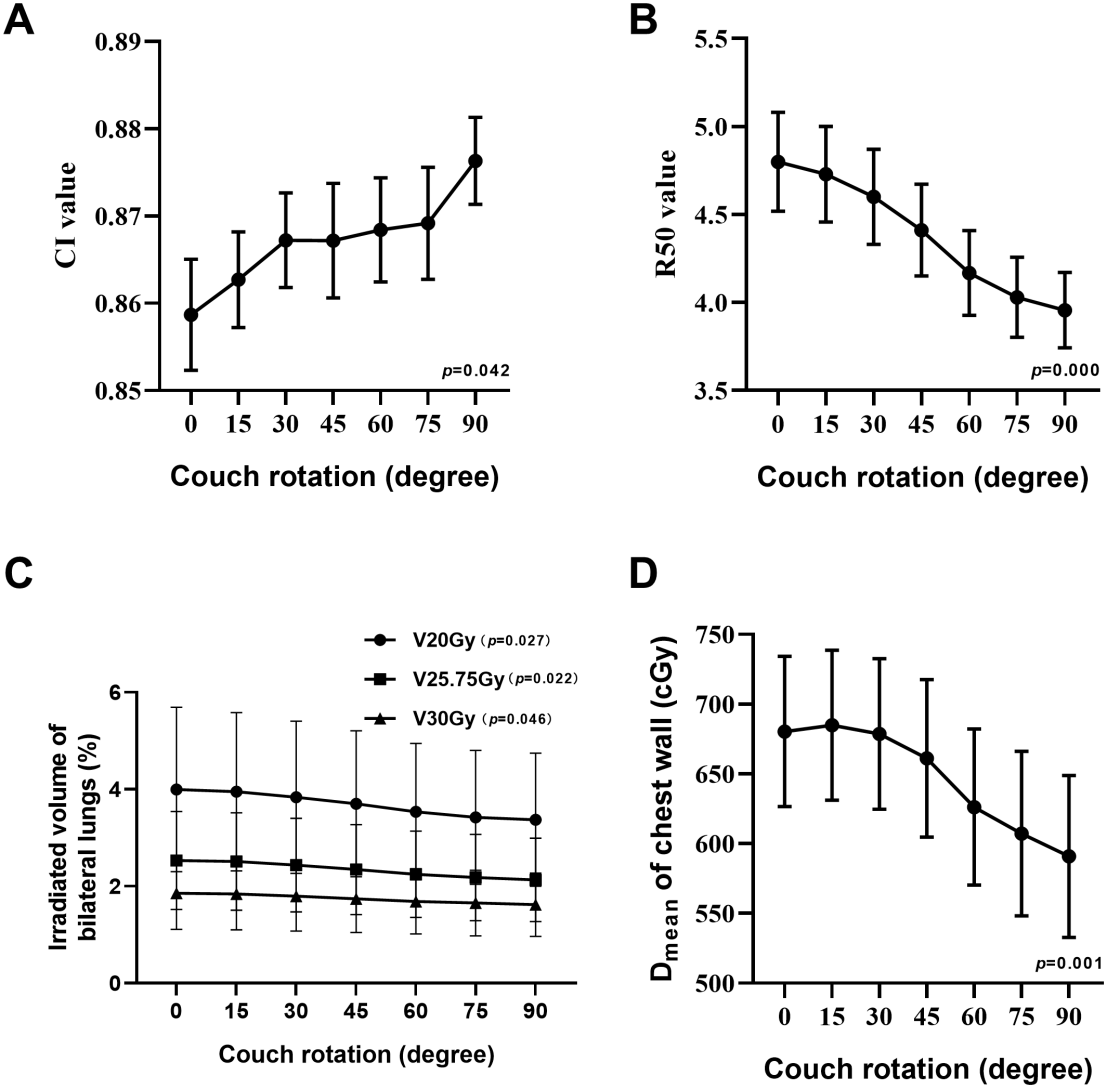
Line charts show the trend change at CI value **(A)**, R50 value **(B)**, V20Gy, V25.75Gy, and V30Gy of irradiated volume of bilateral lungs **(C)** and D_mean_ of chest wall **(D)**.

A summary of plan quality evaluations for various couch rotation angles is shown in **Table 5**. The mean PTV size of 24 patients was 27.11 ± 21.52 cc. In general, as the couch rotation angle increased, all evaluated parameters varied significantly. When comparing C0 to a 90°couch rotation, Dmax, Dmean, and HI decreased from 6728.35 ± 209.56 cGy, 5743.04 ± 93.45 cGy, and 0.281 ± 0.032 to 6500.48 ± 225.26 cGy, 5654.81 ± 109.23 cGy, and 0.245 ± 0.031, respectively. CI increased from 0.859 ± 0.031 to 0.876 ± 0.024 (**Figure 2A**). The HI of ncVMAT plans was lower and closer to 0 compared to cVMAT plans, which had the highest HI (0.281 ± 0.032) (p = 0.000). Similarly, the CI of ncVMAT plans was closer to 1 compared to cVMAT plans, which had the lowest CI (0.859 ± 0.031) (p = 0.042). Additionally, R50% decreased significantly with larger rotation angles (p = 0.000), with incremental reductions of 1.4%, 4.9%, 9%, 13.5%, 16.8%, and 18.4% as the couch rotation increased (**Figure 2B**).

**Table 5:** Plan quality evaluation in VMAT for various couch rotation angles.

	C_0_	NC_15_	NC_30_	NC_45_	NC_60_	NC_75_	NC_90_	*p-value*
D_max_ (cGy)	6728.35 ± 209.56	6726.12 ± 150.43	6713.35 ± 167.21	6654.85 ± 185.40	6605.97 ± 188.81	6562.00 ± 192.36	6500.48 ± 225.26	.000
D_mean_ (cGy)	5743.04 ± 93.45	5731.09 ± 77.75	5735.83 ± 76.31	5719.63 ± 75.78	5699.63 ± 101.47	5680.09 ± 98.01	5654.81 ± 109.23	.000
HI	0.281 ± 0.032	0.276 ± 0.025	0.276 ± 0.026	0.267 ± 0.029	0.262 ± 0.032	0.254 ± 0.031	0.245 ± 0.031	.000
CI	0.859 ± 0.031	0.863 ± 0.027	0.867 ± 0.027	0.867 ± 0.032	0.868 ± 0.029	0.869 ± 0.031	0.876 ± 0.024	.042
R50%	4.88 ± 0.89	4.81 ± 0.86	4.64 ± 0.83	4.44 ± 0.79	4.22 ± 0.74	4.06 ± 0.69	3.98 ± 0.66	.000

A comparison of dosimetry parameters for the OARs is presented in **Table 6**. As couch rotation angle increased, the V20Gy, V25.75Gy, and V30Gy values for the ipsilateral and bilateral lungs showed slight reductions. The C0 plan resulted in the highest V20Gy, V25.75Gy, and V30Gy values, while the NC90 plan produced the lowest values (**Figure 2C**). For the contralateral lung, the V10Gy was lower in the ncVMAT plans compared to the cVMAT plan, which exhibited the highest V10Gy value (0.48 ± 0.68), with p-values less than 0.05 indicating a statistically significant difference among the plans. Additionally, the Dmean to the chest wall demonstrated a statistically significant difference (p = 0.001) (**Figure 2D**), with the NC90 plan achieving the lowest mean dose (590.88 ± 284.00 cGy), a reduction of 13.2% compared to the C0 plan (680.36 ± 263.89 cGy). However, the C0, NC15, and NC30 plans yielded similar Dmean values when the couch rotation was less than 45° (680.36 ± 263.89 cGy for C0, 684.85 ± 263.60 cGy for NC15, and 678.63 ± 264.45 cGy for NC30). For other OARs, no significant differences were observed among the plans, with values remaining comparable across all configurations.

**Table 6:** Dosimetric comparison for organs at risk in VMAT for various couch rotation angle.

		C_0_	NC_15_	NC_30_	NC_45_	NC_60_	NC_75_	NC_90_	*p-value*
Ipsilateral lung	D_mean_(cGy)	555.09 ± 201.82	561.48 ± 206.20	563.59 ± 209.79	572.23 ± 209.08	585.08 ± 206.00	594.07 ± 202.24	591.07 ± 194.88	.192
	V_5Gy_ (%)	27.12 ± 9.55	27.89 ± 10.33	28.65 ± 11.03	30.00 ± 11.37	31.10 ± 11.22	31.61 ± 10.86	31.69 ± 10.65	.021
	V_10 Gy_ (%)	19.01 ± 8.26	19.30 ± 8.39	19.09 ± 8.56	18.94 ± 8.21	19.10 ± 8.16	19.34 ± 8.46	19.21 ± 8.01	.931
	V_20 Gy_ (%)	8.87 ± 4.05	8.76 ± 3.91	8.50 ± 3.74	8.20 ± 3.60	7.86 ± 3.46	7.60 ± 3.41	7.50 ± 3.41	.033
	V_25.75 Gy_ (%)	5.64 ± 2.52	5.58 ± 2.48	5.42 ± 2.40	5.22 ± 2.32	5.00 ± 2.24	4.86 ± 2.24	4.75 ± 2.17	.011
	V_30 Gy_ (%)	4.14 ± 1.88	4.10 ± 1.86	4.00 ± 1.82	3.88 ± 1.76	3.76 ± 1.71	3.69 ± 1.71	3.62 ± 1.66	.043
Contralateral lung	D_mean_(cGy)	122.78 ± 54.51	121.18 ± 53.87	115.40 ± 50.85	110.33 ± 47.53	109.36 ± 47.16	110.16 ± 47.65	109.80 ± 49.83	.180
	V_5 Gy_ (%)	5.72 ± 5.03	5.31 ± 4.69	4.53 ± 4.22	4.06 ± 3.63	4.44 ± 3.79	4.56 ± 3.97	4.32 ± 3.81	.506
	V_10 Gy_ (%)	0.48 ± 0.68	0.37 ± 0.53	0.21 ± 0.45	0.22 ± 0.55	0.19 ± 0.41	0.24 ± 0.44	0.23 ± 0.66	.048
bilateral lungs	D_mean_(cGy)	317.82 ± 109.39	319.48 ± 110.52	317.22 ± 110.45	318.57 ± 109.11	324.39 ± 108.63	329.22 ± 108.00	328.09 ± 106.95	.535
	V_5Gy_ (%)	15.38 ± 5.87	15.48 ± 6.06	15.37 ± 6.16	15.74 ± 6.19	16.49 ± 6.29	16.80 ± 6.34	16.73 ± 6.39	.249
	V_10 Gy_ (%)	8.89 ± 3.75	8.94 ± 3.73	8.75 ± 3.72	8.71 ± 3.71	8.78 ± 3.74	8.93 ± 3.92	8.88 ± 3.80	.914
	V_20 Gy_ (%)	4.00 ± 1.70	3.95 ± 1.63	3.84 ± 1.57	3.70 ± 1.51	3.54 ± 1.41	3.42 ± 1.38	3.37 ± 1.37	.027
	V_25.75 Gy_ (%)	2.53 ± 1.01	2.51 ± 1.00	2.44 ± 0.97	2.35 ± 0.93	2.25 ± 0.89	2.18 ± 0.89	2.13 ± 0.86	.022
	V_30 Gy_ (%)	1.86 ± 0.75	1.84 ± 0.74	1.80 ± 0.72	1.74 ± 0.69	1.69 ± 0.67	1.66 ± 0.68	1.62 ± 0.65	.046
Esophagus	D_max_(cGy)	1622.33 ± 935.90	1652.61 ± 950.80	1620.20 ± 934.20	1544.11 ± 914.59	1511.14 ± 863.87	1451.93 ± 845.03	1486.01 ± 905.80	.353
	D_mean_(cGy)	226.33 ± 141.67	221.94 ± 133.81	223.43 ± 136.68	217.93 ± 131.56	206.02 ± 133.24	211.46 ± 137.57	211.11 ± 137.55	.447
Trachea	D_max_(cGy)	1844.53 ± 1561.82	1889.93 ± 1620.58	1863.93 ± 1597.56	1787.18 ± 1572.34	1750.20 ± 1556.70	1718.72 ± 1542.07	1730.75 ± 1585.17	.416
	D_mean_(cGy)	264.05 ± 211.17	268.48 ± 222.68	269.92 ± 230.09	259.38 ± 212.95	236.58 ± 202.51	237.21 ± 189.94	238.30 ± 190.39	.402
Chest wall	D_max_(cGy)	4103.08 ± 1433.77	4118.49 ± 1473.67	4090.27 ± 1489.77	4050.30 ± 1495.02	4063.95 ± 1513.06	4043.11 ± 1509.48	4048.52 ± 1546.32	.541
	D_mean_(cGy)	680.36 ± 263.89	684.85 ± 263.60	678.63 ± 264.45	661.14 ± 276.78	626.17 ± 273.99	607.24 ± 288.92	590.88 ± 284.00	.001
Heart	D_max_(cGy)	1526.90 ± 2121.60	1550.34 ± 2107.12	1704.50 ± 2040.73	1841.78 ± 2014.48	1878.09 ± 1968.51	1889.87 ± 1919.87	1910.66 ± 1980.86	.030
	D_mean_(cGy)	174.97 ± 249.63	172.08 ± 247.92	167.48 ± 231.90	169.23 ± 220.11	170.18 ± 205.84	168.51 ± 207.53	174.76 ± 211.51	.187
Spinal Cord	D_max_(cGy)	1119.30 ± 512.14	1181.64 ± 534.88	1119.61 ± 534.84	1089.61 ± 505.05	1013.06 ± 496.05	969.79 ± 496.26	1020.13 ± 512.92	.156
	D_mean_(cGy)	135.46 ± 81.53	136.30 ± 84.41	134.63 ± 84.00	136.40 ± 87.87	119.60 ± 77.26	110.81 ± 82.12	114.09 ± 77.86	.099

The plan complexity-related metrics included MD, MU, and DT values for the plans are shown in **Table 7**. The p-values for MD, MU, and DT were all <0.001, indicating statistically significant differences. As the couch rotation angle increased, MD, MU, and DT also increased. When the couch was rotated to 90°, MU increased by approximately 29.9%, from 4503.8 ± 1110.2 to 5815.9 ± 1116.6, and delivery time increased by approximately 22.6%, from 408.5 ± 95.3 seconds to 500.9 ± 93.3 seconds, compared to the C0 values. The gamma index results for all plans demonstrated a high pass rate, exceeding 90% for the 2%/2mm criteria.

**Table 7:** Comparison of plan complexity-related metrics in VMAT for various couch rotation angles.

	C_0_	NC_15_	NC_30_	NC_45_	NC_60_	NC_75_	NC_90_	*p-*value
MD	2.98 ± 0.59	3.08 ± 0.58	3.25 ± 0.55	3.46 ± 0.58	3.63 ± 0.64	3.75 ± 0.64	3.94 ± 0.81	.000
MU	4503.8 ± 1110.2	4712.2 ± 1110.8	4950.5 ± 1237.1	5134.6 ± 1182.1	5341.9 ± 1173.1	5541.5 ± 1140.0	5815.9 ± 1116.6	.000
DT (s)	408.5 ± 95.3	408.5 ± 93.5	431.0 ± 103.5	445.4 ± 98.6	462.6 ± 98.7	477.5 ± 95.8	500.9 ± 93.3	.000

MD, modulation degree; MU, monitor units; DT, delivery time.

## Discussion

SBRT, characterized by large single dose, fewer fractions, a high biological equivalent dose, and a rapid dose fall-off outside the target area, has been recognized as a standard treatment modality for medically inoperable early-stage non-small cell lung cancer (NSCLC) in the NCCN guidelines since 2012 (21). Non-coplanar radiation therapy enhances dose distribution to the target area and adjacent organs at risk by utilizing multiple beam angles and arcs achieved through couch rotation. Lincoln et al. (22) demonstrated that non-coplanar SBRT plans for early-stage NSCLC offered superior protection to surrounding organs while maintaining target conformity. Similarly, Kim et al. (23) reported that the homogeneity index (HI) and conformity index (CI) were superior in ncVMAT compared to cVMAT. Additionally, Hamilton et al. (24) confirmed the advantage of ncVMAT over cVMAT in optimizing dose distribution for lung SBRT. Our study observed similar results for both cVMAT and ncVMAT. Furthermore, we investigated the impact of couch rotation angles in ncVMAT to further optimize dose distribution.

In this study, we assessed the dosimetric benefits and robustness of treatment plans for lung cancer patients by comparing various couch rotations in ncVMAT plans. Our results demonstrated that the average values of Dmax, Dmean, and HI decreased as the couch rotation angle increased, with significant differences observed at couch angles of 45°, 60°, 75°, and 90°. A lower HI value indicates a more uniform dose distribution within the target area, whereas a higher HI value may suggest the presence of dose hotspots or uneven dose distribution. However, in SBRT planning, due to the pursuit of high-dose objectives, a certain degree of dose hotspots is often acceptable, and thus HI may not be as strictly emphasized as in conventional radiotherapy planning. Nonetheless, HI remains an important parameter, especially when there is a need to balance dose hotspots with the protection of surrounding normal tissues. The CI of ncVMAT plans was closer to 1 compared to cVMAT plans, indicating better conformity. In SBRT planning, in addition to ensuring target coverage and conformity, the rate of dose fall-off outside the target area is also a critical focus. According to RTOG 0813 guidelines, the minor deviation and the none deviation of R50% are less than 4.5 and 5.5 for a PTV of 22cc, while 4.3 and 5.3 for a PTV of 22cc (15). In this study, the PTV of the enrolled patients was 27.11 ± 21.52 cc, and the R50% values for all plans were within the minor protocol deviation limits. According to linear interpolation, the none deviation should be less than 4.41. Moreover, when the couch rotation exceeded 45°, R50% values were consistently below 4.41. Overall, ncVMAT plans demonstrated superior quality compared to cVMAT. As couch rotation increased, ncVMAT exhibited improved conformity, homogeneity, and dose distribution.

Radiation-induced lung injury (RILI) is the most common lung toxicity associated with radiation therapy, with an incidence rate of 5-20% (25). Many studies reported that various risk factors were associated with RILI, such as V5 and V20. Interestingly, Jiao Y et al. reported the lung BED volume parameter was a potential reliable value to predict RILI occurrence, they indicated that maintaining lung V_BED70_ below 2.22% in lung cancer patients treated with SBRT reduces the incidence of RILI (26). Consequently, we calculated the BED70 (using an α/β ratio of 10 Gy) in our plan to be 25.75 Gy and found that lung V25.75Gy (%) remained below 2.22% when couch rotation exceeded 60°, suggesting that couch rotations over 60° may be a novel factor in reducing RILI in lung cancer SBRT. ncVMAT has been shown to reduce the dose to OARs by minimizing geometric overlap. In our study, changes in couch rotation angles resulted in dosimetric variations. One possible explanation for this is the spatial repositioning of the tumor relative to the surrounding anatomy due to couch rotation, which also alters the photon path length from normal tissues to the target. Compared to cVMAT, ncVMAT utilizes multi-arc irradiation, delivering doses more efficiently across a 4π solid angle (27).

Chest wall pain and rib fractures are the most common chest wall toxicities following lung SBRT (28). Stephans et al. identified a correlation between tumor and treatment factors with late chest wall toxicity, showing that chest wall dosimetry is associated with the risk of these toxicities (29). Additionally, Shao et al. demonstrated that non-coplanar SBRT for patients with early-stage peripheral non-small cell lung cancer could reduce the dose to the chest wall (30). Yu et al. also found that the mean chest wall V30 and V40 decreased significantly in ncVMAT plans with 15° or 345° couch rotations compared to standard VMAT plans (13). In the present study, the C0, NC15, and NC30 plans showed similar dose distributions for Dmean. However, in ncVMAT plans with couch rotations greater than 45°, the chest wall received the lowest mean dose. As couch rotation increased, the mean dose to the chest wall further decreased. Dunlap et al. reported that a chest wall volume receiving 30 Gy over 35 cc increases the risk of chest wall pain and/or rib fractures (31). Thus, larger couch rotations in ncVMAT may be advantageous in mitigating chest wall radiation toxicity in lung SBRT.

Previous literature has showed no significant dosimetric difference between ncVMAT and cVMAT when the couch rotation angle separation was limited to ±15° (14). However, there are few studies on the dosimetric impact of smaller couch angle separations. Moreover, while the couch rotation setting in ncVMAT are beneficial for lung cancer SBRT, their practical feasibility in clinical treatment remains unclear. This study aims to explore an optimal range for couch rotation angles. To ensure the reliability of the result, all plans were optimized and calculated using the XVMC algorithm, which is accurate within 1% dosimetric uncertainty. The non-coplanar arc gantry rotation was set from -45° to 45° to avoid collision. Variations in dose distributions may be attributed to systematic errors in the IMRT machine, so periodic quality assurance and quality control procedures were performed, including checks on the gantry, collimator, couch, isocenter position, and dose calibration.

However, there are several limitations in this study. The sample size was small, and the findings need to be validated in a larger cohort. Additionally, only patients with a single tumor were included. There are also limitations for the clinical application of ncVMAT. First, as couch rotation increased from 0° to 90°, the plan’s modulation degree (MD) and monitor units (MU) increased by approximately 32.2% and 29.9%, respectively, while the delivery time (DT) was extended by 22.6%. This increase in MD, MU, and DT indicates reduced beam efficiency, which may contribute to increased wear and tear on the equipment. However, considering the setup time for SBRT at our department is about 20-30 minutes, the increase in delivery time (approximately 100 seconds) is relatively short. Secondly, ncVMAT may reduce operational efficiency and introduce angular deviations in couch positioning. Third, rotating the couch increases the risk of collisions with the treatment machine or the patient.

In conclusion, compared to cVMAT, ncVMAT employs more non-coplanar beams through couch rotation, resulting in superior plan quality, including improved homogeneity, greater conformity, and a more compact dose distribution. This approach demonstrated better adherence to the dose distribution criteria outlined in the RTOG 0813 protocol. Our study provides theoretical support for selecting ncVMAT plans in lung cancer SBRT and offers empirical data to guide the determination of optimal couch rotation angles for these plans.

## Data Availability

The original contributions presented in the study are included in the article/supplementary material. Further inquiries can be directed to the corresponding authors.
